# Bridging east and west: integrative approaches to obesity management through traditional Chinese medicine

**DOI:** 10.3389/fendo.2025.1644381

**Published:** 2025-09-17

**Authors:** Qianrong Li, Zhao Liu, Xiaolin Zhang

**Affiliations:** ^1^ Department of Preventive Medicine, Baiyun Hospital of The First Affiliated Hospital of Guangzhou University of Chinese Medicine, Guangzhou, Guangdong, China; ^2^ Department of Endocrinology, Putuo Hospital, Shanghai University of Traditional Chinese Medicine, Shanghai, China

**Keywords:** obesity, wc, WHR, TCM, energy metabolism

## Abstract

This study provides a comprehensive review of research pertaining to the treatment of obesity using traditional Chinese medicine (TCM). It encompasses fundamental theories, epidemiological data, pathological mechanisms, clinical practices, technological advancements, and directions for future research. In the section on fundamental theories, the etiology, pathogenesis, historical development, and theoretical foundations of obesity within TCM are examined. The current state of obesity has been analyzed through an epidemiological lens, along with the application of TCM and relevant clinical research findings. The pathological mechanism section evaluates the effect of TCM on metabolic regulation, inflammatory responses, and adipocyte functionality in the context of obesity. Clinical practices are illustrated through case studies of commonly utilized TCM prescriptions, acupuncture, moxibustion therapy, and integrative approaches combining traditional Chinese and Western medicine. The Technological Advancements section discusses the emergence of new formulations, modern diagnostic technologies, and personalized treatment strategies. The future outlook addresses potential research trajectories, prospects for integrating TCM with contemporary medical practices, and trends in international collaborations. Evidence suggests that TCM holds promise in various aspects of obesity treatment. However, further investigation is necessary to elucidate its efficacy and underlying mechanisms, enhance international cooperation and standardization efforts, and foster the advancement of TCM in obesity management.

## 1 Introduction

The global obesity epidemic has exhibited a concerning trajectory with its prevalence and impact expanding extensively across populations worldwide. Empirical evidence indicates that obesity rates have increased in most regions since the 1990s. According to data published by the World Health Organization (WHO) in 2023, over one billion adults globally are affected by obesity, comprising approximately 430 million men and 570 million women. Notably, the prevalence of obesity has nearly doubled since 2000 (1). In high-income countries, adult obesity rates generally exceed 25% (2). In the United States, the overall obesity prevalence has steadily increased since 1999, notwithstanding a brief plateau between 2009 and 2012, followed by continued growth. For instance, between 2015 and 2016, obesity rates reached 38.0% in adult men and 41.5% in adult women (3).

The obesity epidemic is particularly pronounced in low-income and middle-income countries. Economic development and lifestyle changes have precipitated a “nutritional transition” in many developing nations, characterized by a shift from traditional dietary patterns to the consumption of high-calorie, high-fat processed foods, alongside reduced physical activity. This transition has led to a rapid increase in obesity prevalence over the past few decades. For example, in China, the prevalence of obesity in adults has escalated from 3.3% in 2004 to 16.4% in 2022, with adolescent obesity rates exceeding 20%, positioning China among the countries with the highest obesity burden globally (4).

From an age-related perspective, obesity is no longer confined to adults, and its incidence among children and adolescents has become increasingly significant. The global number of obese individuals aged 5–19 years has surged from 11 million in 1975 to 340 million in 2022, representing an almost thirty-fold increase. In developed countries, childhood obesity prevalence typically exceeds 15%, while in emerging economies, the rate of increase in this demographic outcome is observed in adults (5).

Sex disparities are evident during obesity epidemics. In most countries, women exhibit a higher obesity prevalence than men, particularly in regions such as the Middle East, North Africa, and Latin America, where female obesity rates exceed those of males by 10–15 percentage points. These differences may be attributable to the complex interplay of social, cultural, hormonal, and behavioral factors (6).

Regional variations in obesity prevalence were also notable. Certain Pacific island regions, including Micronesia and Polynesia, experience exceptionally high rates of obesity and diabetes, largely owing to the adoption of Western dietary and cultural practices (7). Similarly, in some developing countries, economic growth and lifestyle modifications have contributed to the rapid increase in the prevalence of obesity. For example, among non-pregnant women in Chad, the obesity prevalence varies according to socioeconomic status and geographic region, reflecting distinct patterns of inequality (5). This global epidemic poses significant threats to individual health by elevating the risk of chronic conditions, such as cardiovascular disease and diabetes, while simultaneously imposing substantial burdens on healthcare systems worldwide, underscoring the imperative for effective prevention and control strategies.

Furthermore, data from the Global Burden of Disease Study highlight the increasing prominence of obesity-related health complications, including chronic diseases such as cardiovascular disorders and diabetes (8). These conditions not only detrimentally affect individuals’ quality of life and life expectancy, but also generate considerable economic strain on national healthcare infrastructure (9). Consequently, a comprehensive understanding of obesity prevalence is critical for the formulation of targeted and effective prevention and intervention measures.

In response, the WHO prioritized the reduction of obesity to 2010 levels as a key objective within the Global Action Plan for the Prevention and Control of Non-Communicable Diseases 2013–2020 (10). Current health guidelines emphasize that obesity fundamentally results from an energy imbalance between calorie intake and expenditure. However, individual-level interventions aimed at reducing caloric consumption and increasing physical activity frequently fail to achieve sustained weight loss (11). Without supportive policies across sectors, such as health, agriculture, transportation, urban planning, environment, food processing and marketing, and education, behavioral modifications alone may prove insufficient (12). Accordingly, the WHO advocates the promotion of healthy dietary practices and increased physical activity at the population level through socially implemented policies and behavioral interventions (10).

The biosocial determinants of obesity represent promising targets for future intervention strategies aimed at mitigating obesity burden. Traditional Chinese Medicine (TCM), grounded in the principles of “harmony between Man and Nature” and “syndrome differentiation and treatment,” aligns with the biopsychosocial model of modern medical practice and offers unique advantages in obesity management. Therefore, this review sought to examine the comprehensive treatment of obesity using TCM, informed by the underlying pathophysiological mechanisms of the condition.

## 2 Definition and classification of obesity

The article titled “Definition and Diagnostic Criteria of Clinical Obesity” (13), published in The Lancet in 2025, fundamentally redefined the conceptualization and classification of obesity. Initially recognized as a disease by the WHO in 1948, obesity has recently gained formal recognition as a disease entity by numerous medical organizations and countries (14–16). The current WHO International Classification of Diseases categorizes obesity as a “chronic disease,” assigning it a specific code 5B81. Nonetheless, the classification of obesity as a distinct disease remains a subject of debate in both the medical and broader communities. The designation of obesity as a disease has significant implications for clinical practice, public health policies, and societal perspectives.

Historically, obesity was defined primarily as a health risk condition (17), with body mass index (BMI) serving as the principal diagnostic criterion—specifically, a BMI exceeding 30 kg/m² or greater than 27.5 kg/m² in Asian populations. However, reliance on BMI as a diagnostic measure presents notable limitations (10, 18). BMI fails to differentiate between adipose and lean body mass, and does not account for variations in fat distribution. Consequently, individuals with BMI values within the normal or overweight ranges may possess excess adiposity and an elevated risk profile associated with obesity (19, 20). Conversely, individuals with high bone density or skeletal muscle mass, such as athletes, may be misclassified as obese based on BMI alone (20).

Alternative anthropometric indices, including waist circumference, waist-to-hip ratio (WHR), and weight-to-height ratio, have been proposed to enhance the diagnostic accuracy. However, these measures have inherent limitations (21). Variability in measurement techniques across populations and sexes, as well as their limited capacity to accurately reflect subcutaneous and visceral fat accumulation, constrain their utility (22). The accumulation of subcutaneous and visceral adipose tissue is closely linked to increased metabolic disease risk, with ectopic fat deposits, such as hepatic fat, contributing to adverse health outcomes (23). Incorporating biochemical markers, such as triglyceride levels, along with waist circumference measurements has been suggested as a method to identify excessive visceral and ectopic fat more effectively (24). While this combined approach may improve risk stratification for future cardiovascular diseases, concerns remain regarding its precision in assessing active disease states (25).

According to the 2025 Lancet publication (13), obesity is defined as excess adipose tissue, which may be accompanied by abnormal distribution or dysfunction of adipose tissue. The etiology of obesity is multifactorial and incompletely understood, involving genetic, environmental, psychological, nutritional, and metabolic factors that disrupt the biological mechanisms that regulate adipose tissue mass, distribution, and function. Obesity can precipitate systemic chronic diseases independent of other comorbidities, inducing functional alterations across the body and various organs and tissues, thereby manifesting significant clinical signs, symptoms, and limitations in daily activities.

Preclinical obesity is a chronic systemic condition characterized by excessive and abnormal adiposity that alters tissue, organ, and individual functions. In preclinical obesity, excess fat is present; however, the function of other tissues and organs remains intact. This stage elevates the risk of obesity and multiple noncommunicable diseases, including type 2 diabetes mellitus, cardiovascular disease, certain cancers, and psychiatric disorders. Although BMI remains a valuable screening tool for identifying individuals who may be overweight or exhibit abnormal adiposity, definitive clinical diagnosis requires confirmation through direct measurement of body fat or at least one additional anthropometric criterion employing thresholds adjusted for age, sex, and ethnicity.

The classification system proposed in the 2025 definition (13) categorizes obesity into primary, secondary, and genetic forms, based on etiology. Genetic obesity encompasses recognized hereditary disorders characterized by hyperphagia, aberrant eating behaviors, and early onset of excessive adiposity, typically manifesting in infancy or childhood. Examples include Prader–Willi syndrome, congenital leptin deficiency, and melanocortin 4 receptor mutations. Secondary obesity is associated with various medical conditions, such as Cushing’s syndrome and hypothyroidism, and the use of certain medications including corticosteroids, antidepressants, and antipsychotics. Primary obesity, the most prevalent form of obesity, has an unknown origin.

## 3 Diagnostic techniques for obesity

### 3.1 Criteria for clinical evaluation of obesity

Clinical assessment of obesity is crucial for ensuring accurate diagnosis and guiding appropriate treatment strategies. Currently, the most commonly employed evaluation metrics include body mass index (BMI), waist circumference (WC), and WHR. BMI, calculated as weight in kilograms divided by the square of height in meters, is widely used as a screening tool for obesity (26). However, BMI does not precisely capture the distribution or quantity of body fat, potentially resulting in misclassification in individuals with increased muscle mass or edema (27).

Despite these limitations, BMI remains the standard measure for determining obesity severity. Clinically, BMI thresholds are often used to categorize obesity into Grade 1 (30–34.9 kg/m²), Grade 2 (35–39.9 kg/m²), and Grade 3 (≥40 kg/m²), which inform intervention decisions and insurance coverage for obesity treatments. Importantly, BMI has become integral to the contemporary definition of obesity as it is endorsed by most healthcare providers, medical organizations, and public health agencies for diagnostic purposes. However, this reliance on BMI poses challenges for fully recognizing and accepting obesity as a complex disease.

Several professional bodies, including the American Association of Clinical Endocrinology and the European Association for the Study of Obesity, advocate assessment approaches that emphasize the pathophysiological characteristics of adipose tissue mass, distribution, and function, rather than relying solely on BMI-based criteria to evaluate the health impacts of excess adiposity.

WC and WHR are particularly valuable for assessing central obesity, which is strongly associated with an increased risk of obesity-related complications such as cardiovascular disease. For instance, elevated WC and WHR beyond established thresholds have been linked to a significantly heightened risk of metabolic syndrome across diverse ethnic populations (28). Additionally, emerging indices, such as the Clínica Universidad de Navarra-Body Adiposity Estimator (CUN-BAE), which incorporates BMI, are gaining attention for potentially providing a more accurate reflection of body fat. Nonetheless, further research is required to validate its consistency with traditional measures and clinical utility (29).

A comprehensive evaluation that integrates multiple clinical assessment tools along with individual medical and family histories enhances the precision of obesity diagnosis and risk stratification. Consequently, BMI should primarily serve as a preliminary screening measure for potential obesity, with supplementary assessments of adiposity necessary to confirm the obesity status in clinical settings. Beyond these measures, the adoption of objective and clinically meaningful criteria to define obesity is imperative.

### 3.2 Application of imaging in the diagnosis of obesity

Imaging technologies are integral to the diagnosis of obesity and offer detailed insights into the body fat distribution and organ-specific fat content. Computed tomography (CT) and magnetic resonance imaging (MRI) are widely employed modalities that enable the precise quantification of visceral and subcutaneous fat, thereby providing a critical foundation for evaluating obesity severity and associated health risks. For instance, MRI-based assessment of hepatic fat content facilitates the diagnosis of non-alcoholic fatty liver disease (NAFLD), a condition prevalent among obese individuals (30).

Ultrasonography also serves as a valuable tool for assessing obesity-related parameters such as abdominal subcutaneous fat thickness and hepatic steatosis. Its advantages include ease of operation, absence of ionizing radiation, and suitability for repeated examinations (31). Additionally, positron emission tomography (PET) offers insights into the pathophysiological mechanisms of obesity by detecting the uptake of specific metabolites and evaluating adipose tissue metabolic activity (32). Nonetheless, each imaging modality presents inherent advantages and limitations: CT and PET involve radiation exposure; MRI is associated with higher costs and longer acquisition times; and the accuracy of ultrasonography may be influenced by the operator’s expertise. Consequently, selection of appropriate imaging techniques should be tailored to the patient’s clinical context and specific diagnostic requirements.

Despite the significant role of imaging in obesity diagnosis, the utilization of methods, such as MRI and CT, remains limited in clinical practice. This underutilization is primarily attributable to high costs, constraints imposed by health insurance coverage, and perceptions held by both patients and healthcare providers.

### 3.3 The role of biomarkers in the diagnosis of obesity

Biomarkers offer novel insights and potential tools for obesity diagnosis. Extensive research has been conducted to identify biomarkers closely linked to obesity and related metabolic disorders. For instance, the concentration of insulin-like growth factor binding protein-1 (IGFBP-1) in obese adolescents is markedly lower than that in normal-weight adolescents, exhibiting a negative correlation with indicators, such as waist-to-height ratio and high-sensitivity C-reactive protein. This suggests that IGFBP-1 may serve as a sensitive biomarker of obesity and its associated metabolic complications (33).

Furthermore, certain metabolites and cytokines are implicated in the pathophysiology of obesity. Serum metabolomic analyses of obese canines have revealed distinct differences in metabolite levels relative to normal-weight dogs, including elevated lipid content, cholesterol, and branched-chain amino acids, along with reduced glucose concentrations (34). In human studies, circulating microRNAs miR-21 and miR-146a have been associated with inflammatory states and CD36 expression in obese males, with decreased levels potentially linked to adiposity-induced inflammatory responses (35). The identification of these biomarkers enhances our understanding of the pathogenic mechanisms of obesity and may provide a foundation for early diagnosis and the development of personalized therapeutic strategies.

## 4 Epidemiology

Obesity has emerged as a significant global public health challenge and is characterized by a persistent upward trend and notable regional disparities. Between 1980 and 2013, the global prevalence of adults with BMI ≥ 25 kg/m² increased markedly, rising from 28.8% to 36.9% among men and from 29.8% to 38.0% among women (36). Similarly, obesity rates among children and adolescents have escalated; in 2013, 23.8% of boys and 22.6% of girls in developed countries were classified as overweight or obese, respectively. Developing countries have also experienced increases, with overweight and obesity rates among boys rising from 8.1% to 12.9% and among girls from 8.4% to 13.4% (36). The prevalence of obesity exhibits considerable regional variation. For instance, certain Pacific Island nations, such as Tonga and Samoa, report exceptionally high adult obesity rates, with female obesity exceeding 50% in some cases. Conversely, while the overall obesity rates in parts of Africa remain relatively low, the rate of increase is more rapid. Factors influencing the prevalence of obesity include economic development, lifestyle modifications, and dietary changes. Economic growth is often correlated with reduced physical activity and increased consumption of calorie-dense, high-fat foods, contributing to rising obesity rates (12). Additionally, accelerated urbanization has further exacerbated the spread of obesity (37). A comprehensive understanding of the global distribution of obesity is crucial for developing targeted prevention and control strategies to mitigate the burden of obesity-related diseases.

Obesity is linked to decreased life expectancy and an elevated risk of early-onset chronic diseases (38, 39). Nonetheless, research on the precise molecular pathways and mechanisms connecting obesity and aging remains limited. Both conditions share numerous physiological characteristics, including systemic inflammation, telomere attrition, gut microbiome dysbiosis, mitochondrial dysfunction, impaired nutrient sensing, disrupted intercellular communication, altered protein homeostasis (40–42), cellular senescence (43), and age-associated DNA hypomethylation (44).

The extent to which obesity accelerates biological aging, thereby precipitating early onset of chronic diseases, remains unclear. A recent multi-event case-control study (45), embedded within the San Diego Longitudinal Study, investigated the relationship between prolonged obesity and expression of biochemical aging markers in young adults. This prospective birth cohort study involved Chilean adults aged 28–31 years, with comprehensive health and nutritional data collected since September 1992. These findings indicated that long-term obesity is associated with biochemical markers indicative of accelerated aging, including epigenetic modifications, telomere shortening, chronic inflammation, impaired nutrient sensing, mitochondrial stress, and disrupted cell-to-cell communication. These results suggest that, in young adults, sustained obesity may expedite biological aging processes, thereby contributing to the development of chronic health conditions.

## 5 Genetic and environmental factors of obesity

Obesity arises from the complex interplay between genetic predisposition and environmental factors. Genetic determinants significantly contribute to obesity development, and numerous genes have been identified as being associated with this condition. For instance, research conducted within a Pakistani cohort led to the construction of a genetic risk score (GRS) based on five gene variants including MC4R and BDNF. This study demonstrated that individuals classified as overweight or obese exhibited significantly higher average GRS rankings than those with normal weight, and a notable correlation was observed between the GRS and obesity-related anthropometric measures (46). Similarly, investigations involving a Mexican mestizo population revealed statistically significant associations between genes such as FTO and APOB and obesity-related comorbidities including hypertension and type 2 diabetes (47).

Environmental factors also play a pivotal role in the etiology of obesity. Dietary habits, as a critical environmental component, often involve consumption patterns characterized by high sugar, fat, and caloric contents, which are common contributors to obesity. Empirical evidence indicates that excessive intake of energy-dense foods such as those rich in fructose and fats promotes weight gain and metabolic dysfunction (48). Additionally, lifestyle modifications, including reduced physical activity and increased sedentary behavior, are closely linked to the rising prevalence of obesity. Gut microbiota has emerged as an influential factor in obesity pathogenesis, and variations in diet and living conditions can alter the composition and functionality of gut microbiota, thereby impacting energy metabolism and adipose tissue accumulation (49). Collectively, genetic factors confer susceptibility to obesity, whereas environmental factors predominantly influence the manifestation and progression of obesity.

Moreover, biopsychosocial determinants have been recognized as significant contributors to obesity development and progression (50). Early-life adversity, psychosocial stress, and unsupportive familial environments have been identified as predictors of elevated obesity risk and adverse cardiometabolic outcomes (51–53). Such adversity encompasses a broad spectrum of environmental stressors, including familial and economic stress, impoverished living conditions, maternal depression, and child neglect, all of which have been implicated in obesity risk through their effects on neurobiological systems (54). These stressors are also associated with an increased incidence of chronic diseases later in life (52). However, the mechanistic pathways underlying these associations remain poorly understood.

Several studies have reported a strong positive correlation between early life adversity and leptin levels in middle-aged individuals, along with a modest negative correlation with adiponectin levels during the same period. The CHAMACOS study, focusing on Mexican-American adolescents with obesity, identified a significant inverse relationship between childhood family environment factors, such as sociodemographic status, family health, maternal depression, home learning environment, and adiponectin concentrations at ages 9 and 14 (55). A recent investigation in Chile further corroborated the association between childhood adversity and adiponectin levels (50). Given the recognized immunometabolic functions of adiponectin and its protective role against obesity development, reduced adiponectin levels are considered unfavorable and constitute risk factors for diabetes and metabolic syndrome. The observed negative association between maternal depressive symptoms and adiponectin aligns with the CHAMACOS findings, which highlighted the strongest inverse relationship when early maternal depression was compared with adiponectin levels measured later in life. Additionally, the negative correlation between maternal depressive symptoms and gastrin levels supports evidence from rodent models, suggesting that early life psychological stress enhances gastrin responses (56). These findings collectively imply that diverse childhood stressors may influence adipokine profiles, metabolic hormone regulation, and obesity risk (57, 58).

## 6 Pathophysiological mechanisms of obesity

### 6.1 Relationship between energy metabolism and obesity

Dysregulation of the energy metabolism is a fundamental factor in the pathogenesis of obesity. Under normal physiological conditions, the body maintains energy homeostasis by balancing energy intake with energy expenditure, thereby preserving stable body weight (59). However, chronic excess energy intake relative to expenditure results in the storage of surplus energy as adipose tissue, culminating in weight gain and the onset of obesity (60). Empirical evidence has indicated that various energy substrates exert distinct metabolic effects. For instance, studies involving C57BL/6 mice have demonstrated that diets rich in sugar, fat, or fructose differentially influence body weight and metabolic parameters. Specifically, high-sugar and high-fat diets are associated with pronounced weight gain, hyperglycemia, and insulin resistance, whereas high-fructose diets predominantly increase visceral adiposity, but exert comparatively modest effects on overall body weight and blood glucose levels (48).

Moreover, critical components of energy metabolism, including lipid and glucose metabolic pathways, frequently exhibit abnormalities in obese individuals. Regarding lipid metabolism, obese subjects typically display enhanced lipogenesis coupled with diminished lipolysis, leading to excessive fat accumulation (61). Concurrently, obesity is linked to mitochondrial dysfunction, which impairs cellular energy production and utilization given its role as a cellular energy generator (62). Research has revealed downregulation of mitochondria-associated protein expression in the white adipose tissue of obese individuals, contributing to mitochondrial impairment and subsequent disruption of adipose tissue energy metabolism (63). These interrelated metabolic perturbations collectively facilitate obesity progression.

### 6.2 Endocrine changes associated with obesity

Obesity is frequently associated with several endocrine alterations that significantly influence metabolic homeostasis in the body. Adipose tissue functions as an endocrine organ by secreting various hormones and cytokines including leptin and adiponectin, which are integral to the regulation of energy metabolism and body weight. Leptin, which is produced by adipocytes, typically suppresses appetite and enhances energy expenditure through its interaction with hypothalamic receptors. Nevertheless, in obese individuals, leptin resistance commonly develops; despite elevated circulating leptin levels, its regulatory effects are impaired, leading to ineffective appetite suppression and continued weight gain (64).

Insulin resistance is a prevalent endocrine disturbance in obesity. Excessive accumulation of adipose tissue induces chronic inflammation, which disrupts insulin signaling pathways, diminishes cellular insulin sensitivity, and consequently impairs normal glucose metabolism. Empirical evidence indicates a strong association between insulin resistance and metabolic abnormalities, including dysglycemia and dyslipidemia, in obese adolescents (65). Furthermore, alterations in thyroid hormone concentrations have been observed in obese patients, potentially affecting the basal metabolic rate. These endocrine modifications interact within a complex network and collectively exacerbate the progression of obesity and its associated metabolic complications.

### 6.3 Interaction between inflammation and obesity

The relationship between inflammation and obesity is characterized by multifaceted interplay. Obesity can induce chronic inflammation, as evidenced by the hypertrophy of adipocytes and expansion of adipose tissue in obese individuals, which leads to localized hypoxia and activation of inflammatory signaling pathways. Research has demonstrated Elevated expression levels of inflammatory mediators, including tumor necrosis factor-α (TNF-α) and interleukin-6 (IL-6), have been observed in the adipose tissue of obese subjects (66). These inflammatory cytokines exert effects not only locally within adipose tissue but also systemically by entering the bloodstream, thereby contributing to chronic systemic inflammation and increasing the risk of comorbidities, such as cardiovascular disease and diabetes.

Conversely, inflammation can influence the progression of obesity by disrupting normal adipocyte metabolic function and promoting lipid accumulation. For instance, inflammatory mediators have been shown to suppress adiponectin secretion from adipocytes; given that adiponectin facilitates fatty acid oxidation and enhances insulin sensitivity, diminished secretion exacerbates metabolic dysregulation (66). Furthermore, inflammation may affect central appetite regulation mechanisms, altering feeding behavior, and thereby further exacerbating obesity. This bidirectional and self-perpetuating cycle between inflammation and obesity complicates therapeutic strategies, underscoring the need for integrated approaches that address both inflammatory processes and metabolic dysfunction in the management of obesity.

### 6.4 Endocrine interferon and obesity

In recent years, numerous environmental chemicals have been identified as modulators of interferon activity and classified as endocrine-disrupting chemicals (EDCs) or endocrine disruptors (67). While the majority of mortality-related studies have concentrated on the disruption of reproductive systems through interference with steroid and thyroid hormone actions (67), an increasing body of evidence indicates that certain EDCs also disrupt metabolic regulatory processes and adipocyte function, thereby contributing to the dysregulation of weight homeostasis and the development of obesity (68–70). These particular chemicals have been termed “obesogens” (71, 72).

Obesogenic EDCs promote lipogenesis and lipid accumulation by perturbing lipid homeostasis, leading to weight gain. This effect may be mediated through an increase in adipocyte number, expansion of adipose tissue volume, or modulation of endocrine pathways that govern adipose tissue development. Typically, early developmental exposure results in an increase in adipocyte number, whereas alterations during adulthood predominantly affect adipocyte size (73). Current findings suggest that adipocyte number is largely established by the end of childhood, and increases in adipocyte number during early life are generally permanent (73). This underscores the critical importance of exposure timing to obesogens, as early life alterations may persist into adulthood and are largely irreversible (69). Additional mechanisms by which obesogens exert their effects include modulation of hormones that regulate appetite, satiety, and food preferences; alteration of basal metabolic rate; and shifts in energy balance favoring caloric storage. Furthermore, obesogens may influence insulin sensitivity and lipid metabolism within key endocrine tissues, such as the pancreas, adipose tissue, liver, gastrointestinal tract, brain, and muscle.

At the molecular level, obesogens can interfere with nuclear transcriptional regulators, including nuclear factor kappa B (NF-κB), which governs lipid flux, adipocyte proliferation, and differentiation. Notably, peroxisome proliferator-activated receptors (PPARs) and steroid hormone receptors function as ligand-activated transcription factors. Upon ligand binding, they interact with specific DNA response elements to regulate gene expression. EDCs that bind to these nuclear receptors have the potential to elicit inappropriate biological responses, including promotion of obesity.

### 6.5 Vitamin D and obesity

Between 2013 and 2018, seven meta-analyses investigated the relationship between obesity and vitamin D status. Saneei et al. (74) conducted a meta-analysis of 34 cross-sectional studies, revealing a modest but statistically significant inverse correlation between vitamin D concentration and BMI. This association was evident in both males and females in developed countries, whereas in developing countries, it was observed exclusively in males. Another extensive meta-analysis encompassing 21 population-based studies (75) that included data from 42,024 adults examined the causal interplay between obesity and vitamin D status. Bidirectional Mendelian randomization analyses of large cohorts indicated that obesity may contribute to reduced vitamin D levels; conversely, low vitamin D status may influence obesity.

The primary circulating form of vitamin D, 25-hydroxyvitamin D [25(OH)D], is fat-soluble and distributed across adipose tissue, muscle, and liver, with minor quantities present in other tissues. Serum 25(OH)D concentrations were consistently lower in obese individuals than in those with normal weight, exhibiting an inverse relationship with body weight, BMI, and fat mass. This pattern has been documented among adults and children across diverse geographic regions, including Northern and Southern Europe, Australia, New Zealand, Saudi Arabia, Latin America, and among white, black, and Hispanic populations in the United States (76–79). On average, serum 25(OH)D levels are approximately 20% lower in obese subjects than in their normal-weight counterparts (76–78). Furthermore, the prevalence of 25(OH)D deficiency among obese individuals ranges from 40% to 80% (79). Evidence suggests that reduced serum 25(OH)D is more likely a consequence of obesity than a causative factor. Supporting this, a large-scale genetic study demonstrated that elevated BMI and obesity-predisposing genetic variants were associated with decreased serum 25(OH)D levels, whereas low 25(OH)D levels and related genetic markers exerted minimal influence on obesity risk (75).

**Figure 1:**
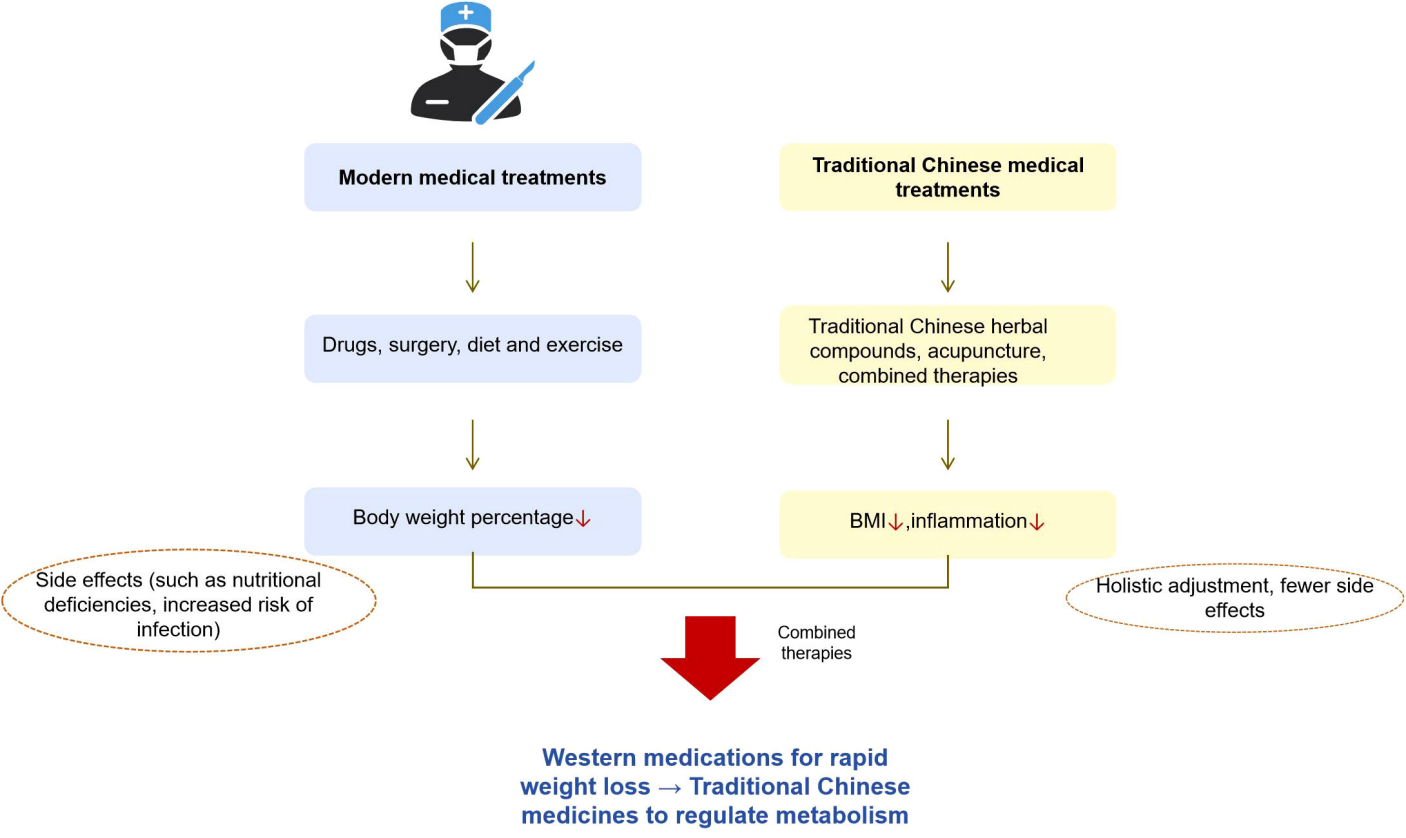
Pathophysiological mechanism network of obesity.

Several mechanisms have been proposed to explain the diminished 25(OH)D levels observed in patients with obesity. The predominant hypothesis posits that increased tissue volume in obesity leads to volumetric dilution of 25(OH)D. Indeed, obese individuals exhibit elevated total amounts of 25(OH)D distributed across the serum, adipose tissue, muscle, and liver, with only a small fraction penetrating other tissues (80). The pathophysiological mechanism of obesity is summarized as follows, see **Figure 1**.

## 7 Modern medical treatment strategies for obesity

### 7.1 The role of diet and exercise intervention in obesity management

Dietary and exercise interventions constitute fundamental approaches for managing obesity. Appropriate modifications in diet can effectively reduce energy intake, whereas consistent physical activity enhances energy expenditure. The synergistic effect of these interventions facilitates the attainment of negative energy balance, thereby promoting weight reduction and metabolic improvement. Numerous studies have investigated the effects of various dietary patterns on various dietary strategies. For instance, low-carbohydrate diets have been shown to induce short-term weight loss; however, their long-term efficacy does not significantly differ from that of other dietary regimens (81).

Exercise intervention also plays a critical role in the management of obesity. Aerobic activities, such as running and swimming, enhance cardiopulmonary function and increase caloric expenditure. Resistance training, including weightlifting, contributes to muscle mass augmentation and elevates basal metabolic rate. Empirical evidence indicates that combining exercise with dietary modifications yields superior outcomes in terms of weight reduction and body composition improvement compared to either intervention alone (82). For example, a study involving obese children demonstrated significant improvements in body weight, body fat percentage, and inflammatory markers following a two-week combined diet and aerobic exercise program (83). Furthermore, such combined interventions have been shown to ameliorate obesity-related sleep disturbances and enhance the quality of life (84). Overall, diet and exercise interventions not only facilitate weight loss but also mitigate obesity-associated metabolic dysfunctions and comorbidities.

Regular physical activity is widely recognized as an optimal strategy for the prevention and management of non-communicable diseases (NCDs) and for promoting general health (85). The WHO guidelines, as outlined by Bull et al. (86), recommend both aerobic and resistance exercises for adults aged ≥ 18 years to achieve health-related benefits. According to the 2020 WHO guidelines, moderate- and vigorous-intensity aerobic activities are defined as physical exertions involving large muscle groups at intensities ranging from 3 to less than 6 and greater than 6 metabolic equivalents (METs), respectively (87, 88). To realize substantial health benefits, adults are advised to engage in 150–300 minutes of moderate-intensity aerobic activity, 75–150 minutes of vigorous-intensity aerobic activity, or an equivalent combination thereof, on a weekly basis. Recent suggestions advocate incorporating training intensity distribution (TID) into exercise planning to enhance precision beyond merely quantifying the total weekly physical activity (89, 90). For overweight and obese women, a bipolar training regimen combining high-intensity interval training with moderate-intensity endurance training, analogous to the TID model employed by athletes, has demonstrated greater efficacy in improving cardiorespiratory fitness than either modality alone, thereby mitigating cardiometabolic risks associated with excess weight (91). Current guidelines also indicate that continuous moderate-intensity continuous training (MICT) sessions lasting 10 min or longer, particularly low-intensity training within a three-zone model, may confer additional benefits (92, 93).

It is important to emphasize that despite the variety of recommended exercise modalities, the paramount consideration for individuals with obesity is the consistent implementation of an exercise program. Identifying a feasible and sustainable form of physical activity tailored to individual preferences and capabilities is crucial for long-term adherence and success.

### 7.2 Advances in the application of drug therapy in obesity

Pharmacotherapy has emerged as a critical component of the comprehensive management of obesity, with an enhanced understanding of the pathophysiological mechanisms underlying obesity. Currently, the United States Food and Drug Administration (FDA) has approved several pharmacological agents for obesity treatment, including orlistat, lorcaserin, liraglutide, and combination therapies such as phentermine/topiramate and naltrexone/bupropion (94). These medications facilitate weight reduction via diverse mechanisms of action. For instance, orlistat promotes weight loss by inhibiting gastrointestinal lipase, thereby reducing fat absorption, whereas liraglutide and similar agents modulate appetite and energy metabolism via their effects on the central nervous system or gastrointestinal hormones (95).

Concurrently, numerous investigational drugs are at various stages of development and offer promising prospects for obesity treatment. Notably, novel glucagon-like peptide-1 (GLP-1) analogs, including semaglutide and its oral formulations, have demonstrated significant efficacy in weight reduction and metabolic improvement in clinical trials (96). Additionally, therapeutic agents targeting alternative pathways, such as melanocortin receptor agonists and neuropeptide Y antagonists, are under investigation and may expand the therapeutic arsenal available for patients with obesity (96). Nonetheless, pharmacotherapy for obesity presents challenges, including adverse effects associated with certain drugs and the need for further evaluation of its long-term safety and efficacy. Therefore, judicious drug selection and vigilant monitoring of patient responses are imperative in clinical practice.

### 7.3 Indication and effect of surgery in the treatment of obesity

Surgical intervention is an effective approach for managing severe obesity, facilitating substantial weight reduction and metabolic enhancement in selected patient cohorts. Predominant bariatric techniques encompass laparoscopic sleeve gastrectomy (LSG) and laparoscopic Roux-en-Y gastric bypass (LRYGB) (97). LSG entails partial resection of the gastric tissue, thereby diminishing gastric volume and restricting food intake to promote weight loss. Empirical evidence indicates that elderly obese patients undergoing LSG exhibit sustained excess weight loss across various postoperative intervals, accompanied by marked improvements in comorbid conditions such as type 2 diabetes, hypertension, and dyslipidemia (98).

Conversely, LRYGB not only limits caloric intake, but also alters nutrient absorption and hormonal secretion through anatomical modification of the gastrointestinal tract, exerting profound metabolic effects. Comparative analyses revealed that LRYGB and LSG combined with duodenojejunal bypass (LSG/DJB) demonstrated superior efficacy in weight reduction compared to LSG alone, particularly in individuals with super morbid obesity (99). Nonetheless, surgical procedures carry inherent risks, including postoperative hemorrhage, infection, and nutritional deficiencies, and are not universally appropriate for obese patients. Consequently, rigorous assessment of surgical indications, considering factors such as body mass index, obesity-related comorbidities, and psychological status, is imperative to optimize patient safety and therapeutic outcomes.

## 8 The basic theory of TCM in treating obesity

### 8.1 Historical evolution of TCM in treating obesity

TCM has a long-standing history in the management of obesity. Ancient texts, such as the Huangdi Neijing, contain pertinent references—for instance, the description of “fat people, the disease of Gao Liang”which acknowledges the association between dietary factors and obesity. Over successive dynasties, treatment approaches have progressively been refined and expanded. Notably, prescriptions from the Ming and Qing Dynasties exemplify the application of syndrome differentiation principles in addressing obesity. Advancements in science and technology have facilitated innovations in TCM obesity treatments while preserving traditional foundations. Specifically, traditional formulations have been subjected to rigorous investigation to elucidate their bioactive constituents and underlying mechanisms. For example, research has demonstrated that certain TCM compounds contribute to weight reduction by modulating signaling pathways involved in lipid metabolism (100). Concurrently, the integration of modern diagnostic techniques has enabled more precise evaluation of TCM efficacy in obesity management (101). Furthermore, clinical research has evolved from anecdotal case reports to large-scale randomized controlled trials (RCTs), thereby generating robust evidence supporting the therapeutic role of TCM in obesity treatment.

### 8.2 Theoretical basis of TCM in the treatment of obesity

TCM presents a distinct theoretical framework for addressing obesity. Central to this framework is the principle of holism, which posits that the human body functions as an integrated body. Consequently, obesity is understood not only as a localized accumulation of adipose tissue but also as a manifestation of systemic dysfunction in fat metabolism. Accordingly, therapeutic interventions emphasize comprehensive regulation, aiming to alleviate obesity symptoms by modulating the functional activities of the internal organs. The cornerstone of TCM treatment lies in syndrome differentiation, whereby individualized treatment regimens are developed based on the patients’ specific clinical presentations, including symptoms, signs, and tongue and pulse diagnostics. Contemporary scientific investigations have elucidated the mechanistic underpinnings of TCM in obesity management. Network pharmacology studies have demonstrated that multiple bioactive constituents within TCM formulations target diverse molecular pathways, thereby influencing fat metabolism, inflammatory responses, and related biological processes. These findings provide a modern scientific rationale that supports the efficacy of TCM in the treatment of obesity (102).

### 8.3 Epidemiological analysis of obesity treated with TCM

Obesity has emerged as a significant global public health challenge, with its annual prevalence increasing. Numerous countries are experiencing an increasing incidence of obesity, which imposes substantial burdens on both society and individuals. In China, shifts in lifestyle and economic development have contributed to an unfavorable trend in obesity rates. Traditional Chinese medicine (TCM) is widely used for the management of obesity. In clinical settings, practitioners often employ TCM prescriptions, acupuncture, and other therapeutic modalities tailored to the patients’ specific conditions. The proportion of obese patients receiving TCM interventions in certain clinics and hospitals has progressively increased. Surveys indicate that, in some regions, a notable segment of obese individuals opt for TCM treatment (103). Additionally, TCM contributes to obesity prevention through approaches such as dietary regulation and traditional physical exercise, which support weight maintenance and reduce obesity risk.

A substantial body of clinical research has substantiated the efficacy of TCM in the treatment of obesity. Systematic reviews and meta-analyses have demonstrated that TCM effectively reduces body weight and BMI. For instance, an analysis of 46 RCTs revealed that TCM outperformed non-pharmacological interventions and placebo or no treatment in lowering body weight and BMI, particularly when the treatment duration was six months or less. Among the therapies assessed, acupotomy was identified as the most effective in achieving these outcomes (104). Furthermore, TCM has been shown to favorably modulate obesity-related biomarkers, including reduction in total cholesterol and triglycerides. It also appears to ameliorate the metabolic syndrome components associated with obesity. Nonetheless, certain limitations exist in the current literature, such as small sample sizes and methodological shortcomings, which diminish the overall quality of the evidence. Consequently, further rigorous, high-quality clinical trials are warranted to definitively establish the efficacy and safety of TCM for obesity management.

Variations in the effectiveness of TCM in obesity have been observed across different demographic groups. Regarding age, adolescent patients undergoing growth and development require TCM interventions that prioritize weight reduction while considering the potential impacts on maturation. Research indicates that combining acupuncture with traditional Chinese medicine effectively decreases BMI and improves insulin resistance in obese adolescents with a favorable safety profile (105). In the adult population, TCM treatments can be individualized based on lifestyle and systemic factors. Elderly obese individuals often present with multiple chronic comorbidities; thus, TCM not only facilitates weight loss but also aids in managing associated chronic conditions. Sex differences are also notable; female obesity is frequently linked to endocrine factors, and TCM offers distinct advantages in regulating female hormonal balance and mitigating obesity. For example, in obese women with polycystic ovary syndrome, TCM has been shown to regulate endocrine function, reduce weight, and enhance ovulation and pregnancy rates (106).

## 9 Pathological mechanism of TCM in the treatment of obesity

### 9.1 Mechanism of TCM on metabolic regulation in obesity

TCM modulates obesity-related metabolic processes through multiple mechanisms. First, in the regulation of lipid metabolism, various constituents of TCM influence lipid synthesis, degradation, and transport. Empirical studies have demonstrated that bioactive compounds within certain TCM formulations can downregulate the expression of genes involved in lipogenesis, while upregulating those associated with lipolysis, thereby mitigating fat accumulation (107). Second, TCM exerts regulatory effects on glucose metabolism. Specific herbal medicines have been shown to enhance insulin sensitivity, facilitate glucose uptake and utilization, and ameliorate insulin resistance in individuals with obesity. For instance, the combination of astragalus and yam has been reported to improve glucose metabolism in type 2 diabetic rat models by modulating relevant molecular targets (108). Furthermore, TCM influences energy metabolism by activating signaling pathways that increase energy expenditure and contribute to weight reduction. Certain TCM compounds have been found to upregulate genes related to mitochondrial oxidative phosphorylation, thereby enhancing mitochondrial function and promoting overall energy metabolism (109).

### 9.2 The effect of TCM on inflammatory response in obesity

**Figure 2:**
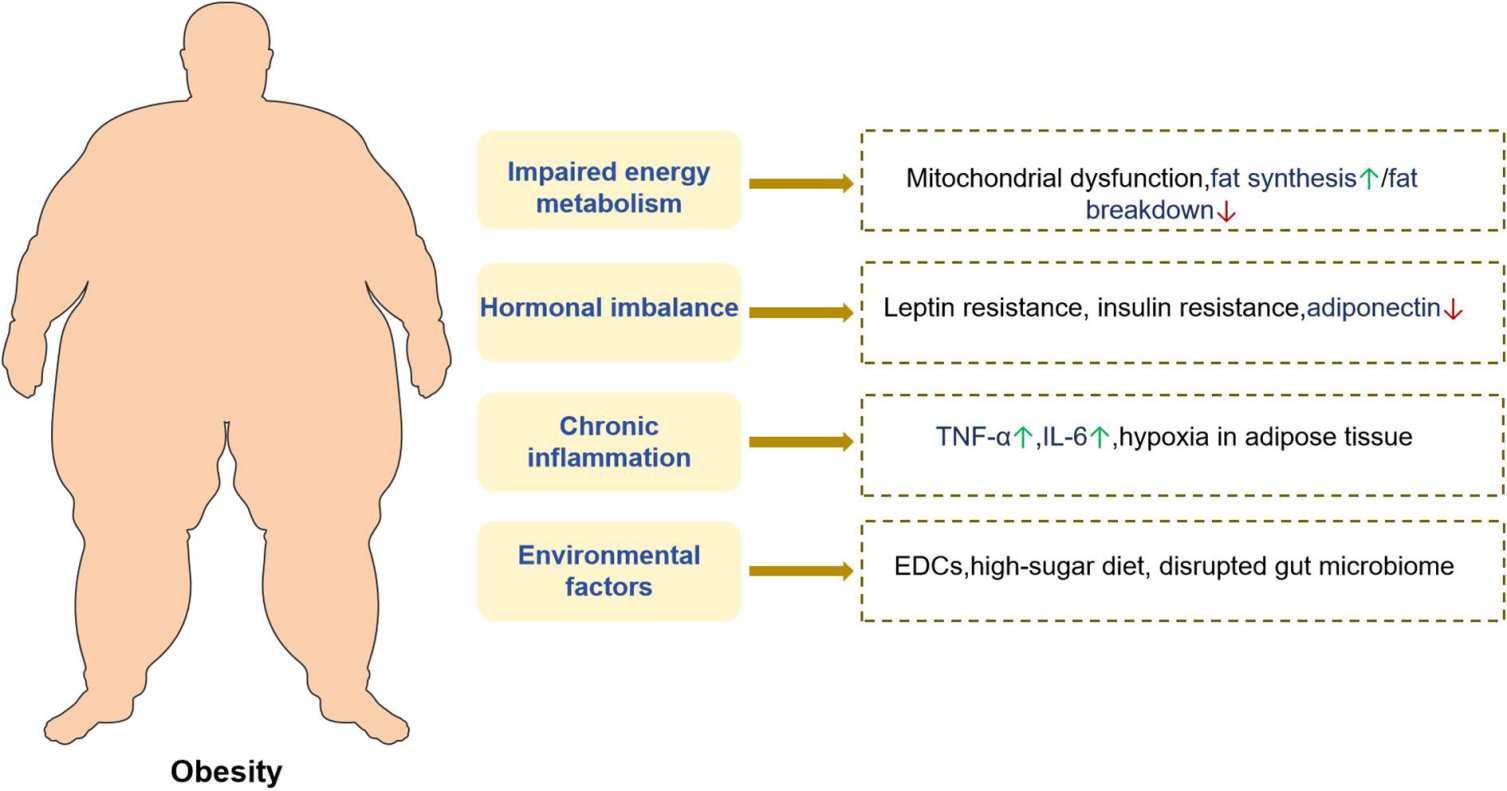
The mechanism and effect of traditional Chinese medicine regulating gut microbiota to improve obesity.

Obesity is frequently linked to chronic inflammation and TCM has been recognized for its significant role in mitigating inflammatory processes. Empirical evidence indicates that certain TCM formulations can effectively reduce the levels of pro-inflammatory cytokines, including TNF-α and IL-6, in obese individuals. For instance, Taeeumjowuitang (TJ) has been demonstrated to attenuate inflammatory responses in high-fat diet-induced obese murine models, with transcriptomic analyses revealing its capacity to modulate the expression of inflammation-related genes in adipose tissue (109). Furthermore, TCM interventions have been shown to alleviate systemic inflammation by modulating the gut microbiota and enhancing intestinal barrier integrity, thereby limiting the translocation of inflammatory agents such as endotoxins into the circulatory system. Several studies have reported that TCM treatment leads to an increase in beneficial gut bacterial populations, a reduction in pathogenic bacteria, improved intestinal barrier function, and a consequent decrease in systemic inflammation in obese patients (110). The mechanism by which TCM regulates the intestinal flora to improve obesity is summarized as follows, as shown in **Figure 2**.

### 9.3 Modulation of adipocyte function by TCM in obesity

TCM has been shown to modulate adipose tissue function in individuals with obesity. First, TCM influences adipocyte proliferation and differentiation. Certain bioactive compounds derived from TCM have been shown to inhibit the maturation of preadipocytes into fully differentiated adipocytes, thereby reducing the adipocyte number. Empirical studies have indicated that specific TCM extracts can downregulate the expression of transcription factors critical for adipocyte differentiation, effectively suppressing this process (111). Second, TCM regulates the metabolic activity of adipocytes by enhancing lipolysis and promoting fatty acid oxidation, which contribute to decreased fat accumulation. For instance, some TCM agents activate lipolytic signaling pathways within adipocytes, facilitating the breakdown and utilization of lipids. Moreover, TCM modulates the secretion of adipocyte-derived cytokines including adiponectin and leptin, which are integral to the regulation of energy homeostasis and body weight management (112).

### 9.4 A modern test for TCM treatment of obesity

Contemporary detection technologies offer enhanced precision for evaluating the efficacy of TCM in the treatment of obesity. Near-infrared spectroscopy enables the monitoring of active ingredient variations during the processing of TCM, thereby ensuring the consistency and quality stability of medicinal products, which is critical for therapeutic effectiveness (113). Metabolomics approaches facilitate the analysis of metabolic alterations in obese patients before and after TCM intervention, thereby elucidating the mechanisms underlying the anti-obesity effects of TCM. For instance, metabolomic investigations have demonstrated that TCM modulates the metabolites associated with energy and lipid metabolism in obese individuals. Furthermore, the integration of network pharmacology and molecular docking techniques allows the prediction of interactions between TCM active compounds and obesity-related molecular targets, providing a theoretical foundation for the development of novel TCM-derived therapeutics. Additionally, advanced imaging modalities, such as MRI, offer direct visualization of adipose tissue distribution and dynamics in obese patients, thereby enabling the assessment of the impact of TCM treatment on fat tissue morphology and volume.

## 10 Clinical practice of TCM in the treatment of obesity

### 10.1 Application of common traditional Chinese medicine prescriptions in the treatment of obesity

Zheng et al. (114) conducted an RCT to investigate the effect of Xinjia Xiaoke Fang on glucose and lipid metabolism in obese individuals diagnosed with type 2 diabetes mellitus. The study enrolled 116 obese patients with type 2 diabetes who were randomly assigned into two groups of equal size (n=58 each): an observation group and a control group. The control group received standard clinical treatment, whereas the observation group received standard treatment in conjunction with the Xinjia Xiaoke Fang formulation. Comparative analyses were performed on the therapeutic outcomes, TCM syndrome scores, glucose and lipid metabolic parameters, and inflammatory markers before and after the intervention. These findings indicated that Xinjia Xiaoke Fang effectively ameliorated abnormal glucose and lipid metabolism and reduced inflammation in obese patients with type 2 diabetes, suggesting its potential for broader clinical application.

Sun Liping et al. (115) designed an RCT to evaluate the effects of Qiwei Baizhu Powder combined with Buyang Huanwu Decoction on glucose and lipid metabolism as well as insulin resistance in obese patients with type 2 diabetes mellitus. A total of 86 obese patients with type 2 diabetes were enrolled and divided into a control group receiving conventional treatment and a study group receiving conventional treatment supplemented with the two herbal formulations. Clinical efficacy, TCM syndrome scores, metabolic indices related to glucose and lipids, and insulin resistance levels were assessed before and after treatment. The results demonstrated that the combined administration of Qiwei Baizhu Powder and Buyang Huanwu Decoction significantly improved glucose and lipid metabolism and contributed to the reduction in insulin resistance in this patient population.

Yang et al. (116) conducted an RCT to assess the clinical efficacy of a modified Cangfu Daotan Decoction in treating patients with simple phlegm-dampness-type obesity accompanied by dyslipidemia. Seventy-two patients meeting these criteria were randomly allocated to the treatment and control groups (n=36 each) using a random number table. The control group received basic treatment plus metformin hydrochloride tablets, whereas the treatment group received the modified Cangfu Daotan Decoction in addition to the control regimen. Both the groups underwent treatment for 12 weeks. Outcome measures included BMI, abdominal circumference, homeostasis model assessment of insulin resistance (HOMA-IR), blood lipid profiles, TCM syndrome scores, and overall clinical efficacy evaluated before and after the intervention. The study concluded that the modified Cangfu Daotan Decoction effectively reduced body weight and waist circumference, regulated lipid metabolism, alleviated insulin resistance, and improved clinical symptoms in patients with simple phlegm-dampness-type obesity and dyslipidemia.

### 10.2 Application and effect of acupuncture and moxibustion therapy in obesity

Wang Yao et al. (117) conducted an RCT to evaluate the efficacy and underlying mechanisms of acupoint catgut embedding in treating obesity accompanied by insulin resistance (IR). Seventy-six patients were randomly assigned to either the control group or the observation group, each comprising 38 participants. The control group received diet and exercise interventions, whereas the observation group received acupoint catgut embedding in addition to these interventions. Outcome measures included body mass index (BMI), waist-to-hip ratio, body fat percentage, serum total cholesterol (TC), triglycerides (TG), fasting plasma glucose (FPG), fasting insulin (FINS), and HOMA-IR. Furthermore, serum levels of PPARγ and NF-κB were assessed. The findings indicated that acupoint catgut embedding significantly reduced BMI, waist-to-hip ratio, and body fat percentage; improved lipid profiles; and ameliorated insulin resistance. These effects were potentially mediated through the modulation of serum PPARγ and NF-κB levels, demonstrating their superiority over diet and exercise alone.

Luo et al. (118) performed an RCT to investigate the impact of acupoint catgut embedding combined with triple energizer conditioning on peripheral serum neuropeptide Y (NPY) and adiponectin (ADP) levels in patients with simple obesity. Fifty-eight obese individuals were randomly allocated to either a catgut-embedding therapy group (n=28) or a Western medicine group receiving metformin (n=30). Post-treatment assessments included BMI, WC, WHR, and serum NPY and ADP concentrations. The results demonstrated that the combined triple energizer regulation and acupoint catgut-embedding approach was more effective than metformin in reducing obesity-related parameters. Additionally, this method favorably modulated serum NPY and ADP levels, suggesting a potential mechanism involving improvement of leptin and insulin resistance, thereby facilitating weight loss.

Qiuzi et al. (119) designed an RCT to examine whether acupoint catgut embedding delayed gastric emptying, suppressed appetite, reduced food intake, decreased lipid absorption, and consequently lower body mass by influencing GLP-1 concentrations in patients with simple obesity characterized by stomach-heat stagnation and spleen deficiency. Sixty patients were randomly divided into an observation group (n=30) receiving acupoint catgut embedding therapy and a control group (n=30) treated with conventional acupuncture. Changes in blood GLP-1 levels and BMI were measured pre- and post-intervention. The study concluded that acupoint catgut embedding effectively inhibited gastric emptying rate, appetite, and eating frequency, leading to BMI reduction in this patient population, potentially through the modulation of circulating GLP-1 concentrations.

### 10.3 Clinical practice of TCM comprehensive treatment for obesity

Zhu Hairui et al. (120) conducted a RCT to evaluate the clinical efficacy of acupuncture combined with the traditional Chinese medicine approach of “Guben Peiyuan, Jianpi Hewei” in treating obese patients with type 2 diabetes mellitus. The study enrolled 80 obese individuals diagnosed with type 2 diabetes who were randomly assigned to two groups of 40 participants each. The control group received metformin hydrochloride tablets in combination with miglitol tablets, whereas the treatment group received the same pharmacological regimen as acupuncture therapy. Both groups were treated for 30 d. Comparative analyses of relevant clinical parameters before and after the intervention revealed that the combined acupuncture and traditional Chinese medicine approach produced significant clinical benefits. Specifically, this method effectively reduced body weight and fat mass, normalized blood glucose levels, and notably improved insulin resistance, as evidenced by increased FINS and high-density lipoprotein cholesterol (HDL-C) levels. In addition, reductions in BMI, FPG, TG, and HOMA-IR were observed. The intervention demonstrated a favorable safety profile with no reported adverse effects, supporting its potential for clinical application.

Zhou et al. (121) designed an RCT to assess the therapeutic effects of Qiwei Baizhu Powder combined with meridian-based abdominal massage in obese patients with simple obesity. A total of 150 individuals diagnosed with simple obesity were enrolled and randomly divided into two groups of 75 participants each. The control group received standard clinical interventions, while the treatment group was administered Qiwei Baizhu Powder alongside abdominal massage targeting meridian pathways, in addition to conventional treatment. The outcomes measured included clinical efficacy, body fat percentage, body mass, body mass index (BMI), glucose and lipid metabolism parameters, leptin, and insulin levels. The findings indicated that the combined intervention significantly decreased body fat percentage, body mass, and BMI, while also modulating glucose and lipid metabolism as well as leptin and insulin concentrations. These results substantiate the definitive therapeutic effect of Qiwei Baizhu Powder combined with meridian abdominal massage in the management of simple obesity.

Han et al. (122) performed an RCT to investigate the clinical efficacy of Jianpi Tiaogan Yin combined with Yiyi Navel acupuncture in treating simple obesity characterized by liver depression and spleen deficiency. The study included 67 patients with this specific subtype of simple obesity who were randomly allocated into three groups: a traditional Chinese medicine group (n=23), an umbilical acupuncture group (n=22), and a combined acupuncture and medicine group (n=22). All the participants received guidance on healthy lifestyle practices. The traditional Chinese medicine group was treated with Jianpi Tiaogan Yin alone. The umbilical acupuncture group underwent acupuncture centered on the navel pistil, targeting specific acupoints (Zhen, Xun, Li, and Kun) three times per week and additional points (Kun, Dui, and Kan) twice per week. The combined group received both Jianpi Tiaogan Yin and Navel acupuncture. Each treatment course lasted seven days, with a total of eight courses administered. After eight weeks, assessments of body mass, BMI, waist-hip ratio (WHR), and four blood lipid parameters were conducted, alongside evaluations of traditional Chinese medicine syndrome scores and adverse events. The combined treatment group exhibited significant reductions in body weight and related anthropometric measures, improved lipid metabolism profiles, and alleviation of the clinical symptoms associated with liver depression and spleen deficiency. These improvements contributed to enhanced patient quality of life, indicating a marked therapeutic benefit of the combined Jianpi Tiaogan Yin and Yiyi Navel acupuncture intervention for this patient population.

### 10.4 The molecular mechanism of junction and its clinical effect

Contemporary investigations into TCM for obesity treatment are transitioning from reliance on traditional experiential summaries toward an advanced phase characterized by the integration and validation of “clinical efficacy–target of action–molecular mechanism.” To meet the biomedical community’s stringent standards for evidence and to genuinely advance the modernization and internationalization of TCM, it is imperative that the molecular mechanisms identified in preclinical research be closely correlated with objective outcomes derived from human clinical trials.

Acupuncture and moxibustion are prevalent nonpharmacological interventions for obesity management. A critical challenge lies in demonstrating that acupuncture’s effects surpass placebo responses and elucidating the underlying biological mechanisms. An RCT conducted by Yao et al. (123) provided robust evidence in this regard. This study not only documented improvements in macroscopic clinical parameters such as BMI and WHR but also identified alterations in key serum molecular markers among obese patients exhibiting IR. Compared to the control group receiving diet and exercise alone, the cohort treated with acupoint catgut embedding exhibited superior weight loss and significant modulation of serum levels of PPARγ and NF-κB. PPARγ functions as a pivotal transcription factor governing adipocyte differentiation and lipid metabolism, whereas NF-κB is central to the inflammatory signaling pathways. The principal contribution of this study lies in its direct demonstration that physical interventions can modulate specific molecular pathways implicated in lipid metabolism and inflammation in humans. This establishes a direct mechanistic link between the clinically observed outcomes (weight reduction, lipid profile improvement, and amelioration of insulin resistance) and the regulation of PPARγ and NF-κB. The study design, which concurrently assesses molecular markers within clinical trials, provides high-quality evidence to elucidate the biological mechanisms underlying TCM therapies.

Da-Chai-Hu-Tang is frequently used in the management of obesity concomitant with metabolic syndrome. Numerous investigations have elucidated multi-targeted mechanisms at both the clinical and fundamental levels. A randomized, development-labeled clinical trial involving high-risk patients receiving statin therapy with residual hypertriglyceridemia demonstrated that Da-Chai-Hu-Tang significantly decreased triglyceride concentrations over a 12-week period, outperforming the comparator OMEGA-3 (124). This definitive clinical efficacy prompted detailed exploration of the underlying molecular mechanisms. An animal study published in Frontiers in Pharmacology (125) offers mechanistic insights, revealing that Da-Chai-Hu-Tang confers protection against drug-induced acute intrahepatic cholestasis via activation of peroxisome proliferator-activated receptor alpha (PPARα). PPARα plays a critical role not only in maintaining bile acid homeostasis but also as a central regulator of lipid and cholesterol metabolism. Furthermore, the study indicated that the protective effects of Da-Chai-Hu-Tang were associated with the modulation of downstream inflammatory signaling pathways, including JNK, IL-6, NF-κB, and STAT3.

Jian Pi Tiao Gan Yin, a traditional Chinese medicinal formulation, has been demonstrated to exert beneficial effects on weight reduction and lipid lowering (126). To systematically elucidate its mechanisms, researchers have employed a high-fat diet (HFD)-induced obesity mouse model, integrating 16S rDNA sequencing with untargeted metabolomics for comprehensive analysis. This study first confirmed the clinical efficacy of Jian Pi Tiao Gan Yin in animal models, showing significant reductions in body weight, TC, TG, and low-density lipoprotein cholesterol (LDL-C) in obese mice. More importantly, the investigation uncovered mechanistic insights: (1) modulation of the gut microbiota, with Jian Pi Tiao Gan Yin significantly altering the intestinal microbial composition and increasing the abundance of beneficial bacterial taxa; and (2) remodeling of fecal metabolic profiles, with metabolomic analyses revealing therapeutic effects linked to the regulation of multiple key metabolic pathways, including linoleic acid metabolism, alpha-linolenic acid metabolism, glycerophospholipid metabolism, and arachidonic acid metabolism. This study integrates randomized controlled trial efficacy with cutting-edge domains of intestinal microecology and host metabolism, proposing that Jian Pi Tiao Gan Yin mediates its anti-obesity effects via the axis of “regulation of intestinal microbiota–remodeling of metabolic pathways.” This not only provides a contemporary biological rationale for the traditional theory of “soothing the liver and strengthening the spleen” but also establishes a scientific foundation for the development of novel obesity therapies grounded in intestinal microecology.

### 10.5 Technical progress of TCM in the treatment of obesity

To enhance the efficacy and convenience of TCM for obesity treatment, the development of novel formulations has emerged as a prominent area of research. First, the integration of nanotechnology offers promising opportunities for advancing TCM preparation. Nanometer-scale TCM formulations exhibited improved solubility, stability, and bioavailability. For instance, nanoformulated TCM can more effectively traverse biological membranes, thereby increasing drug distribution and absorption within the body, which ultimately augments therapeutic outcomes (127). Second, innovation in new dosage forms for TCM compounds, such as sustained-release and targeted delivery systems, is imperative. Sustained-release formulations enable gradual drug release, prolonging the duration of action and reducing dosing frequency. Targeted delivery systems facilitate precise drug action on adipose tissues and other relevant targets, thereby enhancing treatment specificity. Another significant research direction involves the extraction and purification of active constituents from TCM to develop novel preparations characterized by high efficacy and low toxicity. Utilizing modern technological approaches, active ingredients within TCM have been identified, enabling the development of targeted pharmacological interventions for obesity management.

### 10.6 The current status and challenges of TCM in the treatment of obesity

Although numerous clinical studies have asserted that TCM is generally well tolerated and associated with minimal adverse effects in the management of obesity, a thorough examination of the literature and clinical case reports reveals significant safety concerns that warrant attention. These concerns encompass not only the intrinsic toxicity of Chinese herbal medicines but also complex issues related to product quality control and potential drug interactions.

TCM is associated with a broad range of adverse reactions ranging from mild gastrointestinal disturbances to severe organ damage.

1. Hepatotoxicity Liver injury is one of the most serious and frequently reported adverse events associated with Chinese herbal medicines. Several herbs used for weight loss or metabolic regulation have demonstrated potential hepatotoxic effects. For instance, Polygonum multiflorum Thunb, commonly utilized for hair pigmentation and anti-aging purposes, has been implicated in multiple cases of liver injury when improperly administered (128). Similarly, herbs such as Senecio scandens raise safety concerns owing to the presence of hepatotoxic pyrrolizidine alkaloids (129).
2. Nephrotoxicity: Certain TCM components have been documented to impair renal function. A notable example is the use of aristolochic acid-containing herbal preparations, which led to the emergence of “Chinese herbal nephropathy” during weight loss treatments in Europe in the 1990s, resulting in renal failure and, in some cases, urothelial carcinoma (130).
3. Cardiovascular and Neuropsychiatric Risks: Some weight loss-related Chinese herbal medicines, particularly those containing ephedra, have been associated with elevated risks of cardiovascular and psychiatric adverse events, including palpitations, hypertension, anxiety, and insomnia (131).
4. Product Quality and Adulteration: The Insufficient regulatory oversight of the Chinese herbal medicine market has resulted in inconsistent product quality. In pursuit of rapid efficacy, certain weight-loss products have been illicitly adulterated with prohibited Western pharmaceutical agents such as sibutramine and fenfluramine, posing significant health hazards to consumers (130).

**Figure 3:**
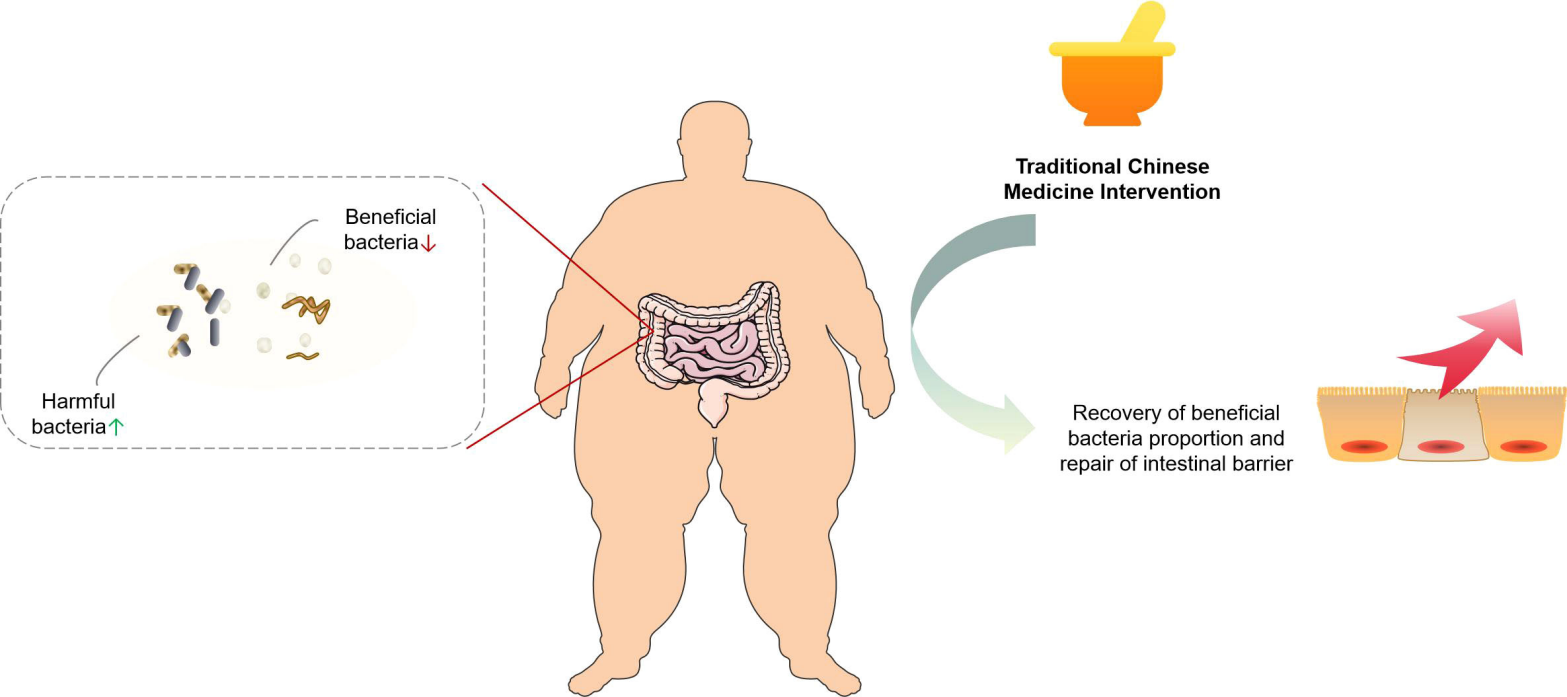
Multi-target mechanisms of traditional Chinese medicine in the treatment of obesity.

Within the integrated medical framework, obese patients frequently receive concurrent treatment with both TCM and Western pharmaceuticals, thereby increasing the potential for drug interactions. The complex composition of TCM formulations can modulate the metabolism and therapeutic effects of western drugs by influencing cytochrome P450 (CYP450) enzyme activity. For example, amiodarone, a drug with a narrow therapeutic index used in cardiovascular disease management, has been shown to alter the pharmacokinetics of fucus extract, a common ingredient in weight-loss supplements, potentially leading to enhanced toxicity or diminished efficacy. This interaction presents a notable risk for obese patients with cardiovascular conditions (132). Nevertheless, the current knowledge regarding the interactions between TCM and commonly co-administered medications for obesity, such as hypoglycemic and antihypertensive agents, remains limited. The multi-target mechanism of TCM in treating obesity is summarized as follows, as shown in **Figure 3**.

## 11 Deficiencies of existing research

Although this article references numerous studies, including clinical trials involving TCM prescriptions, acupuncture and moxibustion therapies, and combined acupuncture and medicinal interventions that have reported favorable therapeutic outcomes, several critical methodological issues generally constrain the robustness of the evidence.

1. Small Sample Size: The majority of RCTs feature relatively small sample sizes, typically ranging from several dozen to just over 100 participants. Such limited sample sizes diminished the statistical power of these studies and increased the likelihood of random errors influencing the results, thereby restricting the generalizability and reproducibility of the findings.
2. Inadequate Randomization and Blinding: Although studies identify themselves as RCTs, they often fail to provide detailed descriptions of randomization procedures, including sequence generation and allocation concealment, which raises concerns regarding potential allocation bias. Moreover, owing to the inherent characteristics of TCM interventions, implementing rigorous double-blinding is challenging. The absence of effective blinding introduces risks of performance and detection biases, potentially leading to an overestimation of the true effects of the intervention.
3. Selection Bias: Clinical studies may have been subject to selection bias during participant recruitment. For instance, the inclusion of specific TCM syndrome types, such as phlegm dampness or liver depression with spleen deficiency, while consistent with TCM clinical practice, results in a highly homogeneous study population. Consequently, the applicability of these findings to other syndrome categories or to a broader population of individuals with obesity is limited.
4. Insufficient Follow-Up Duration: Given that obesity is a chronic condition characterized by frequent relapse, evaluation of sustained therapeutic efficacy is essential. However, existing studies generally feature short intervention and follow-up periods, typically spanning only a few weeks to a few months. This limitation hinders the assessment of long-term treatment effects and the potential for weight regain following the cessation of therapy, thereby constraining conclusions regarding the enduring value of TCM interventions in chronic disease management.

## 12 Future prospects of TCM in the treatment of obesity

### 12.1 Potential research direction of traditional Chinese medicine in treating obesity

Future research on obesity treatment using TCM can be pursued through multiple avenues. First, an in-depth investigation of the relationship between TCM-mediated regulation of gut microbiota and obesity is warranted. Although existing studies indicate that TCM can modulate the intestinal flora to ameliorate obesity, the precise mechanisms and key microbial taxa involved remain to be elucidated. Employing advanced techniques, such as metagenomics and metabolomics, to comprehensively analyze the impact of TCM on gut microbiota and its metabolites will provide a foundation for the development of obesity therapeutics targeting microbial regulation. Second, it is imperative to enhance research on the multitarget mechanisms of TCM. Utilizing approaches such as network pharmacology and systems biology can facilitate the exploration of synergistic interactions between various TCM components and their effects on obesity-related signaling pathways, thereby offering theoretical support for the optimization of TCM formulations. Furthermore, investigating the integrative application of TCM in combination with other interventions, such as exercise and dietary therapies, represents a significant direction for formulating more effective and comprehensive treatment strategies.

### 12.2 The prospect of treating obesity by combining traditional Chinese medicine with modern medicine

**Figure 4:**
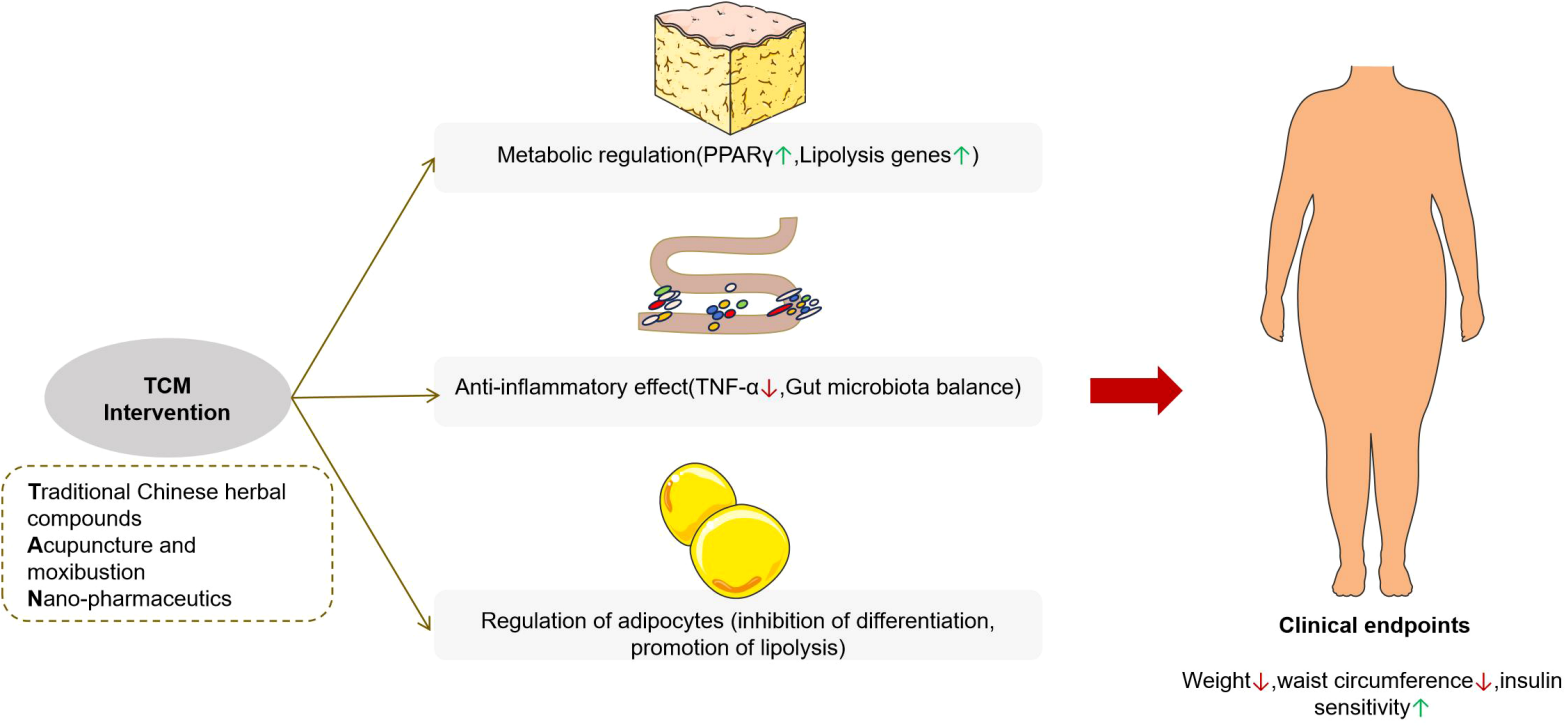
Modern medicine and traditional Chinese medicine in the treatment of obesity.

The integration of TCM and modern western medicine presents promising prospects for the treatment of obesity. Modern Western medicine offers precision and objectivity in the diagnosis and assessment of obesity, whereas TCM provides distinctive advantages in holistic regulation, symptom improvement, and the mitigation of adverse effects. A synergistic combination of these two approaches can leverage their complementary strengths. For instance, in pharmacological interventions for obesity, Western medicine facilitates rapid weight reduction, whereas TCM contributes to metabolic regulation, amelioration of associated complications, and attenuation of side effects induced by Western treatments. Furthermore, the application of advanced modern medical technologies, such as genetic testing and imaging modalities, enables more accurate evaluation of the efficacy and underlying mechanisms of TCM. Additionally, evidence from multicenter, large-scale clinical trials has substantiated the effectiveness and safety of integrated TCM and Western medicine approaches in obesity management. This evidence supports the clinical adoption of integrative therapies and promotes their establishment as mainstream treatment paradigms for obesity. The mechanism of combining TCM with modern medicine is summarized as follows, as shown in **Figure 4**.

### 12.3 International cooperation and development trend of traditional Chinese medicine in treating obesity

Amid growing global concerns regarding health issues, international collaboration in the application of TCM for obesity treatment has notably expanded. Various countries have engaged in exchanges and cooperative efforts encompassing both the research and clinical practice of TCM, collectively seeking effective approaches for managing obesity through traditional methods. For instance, multinational research teams have conducted joint clinical trials investigating TCM compounds for obesity treatment, facilitating the sharing of research findings and clinical experience. The development of TCM-based obesity therapies is expected to emphasize greater standardization. The establishment of unified diagnostic criteria, treatment protocols, and efficacy evaluation systems is anticipated to enhance international acceptance and utilization of TCM. Concurrently, leveraging modern scientific and technological tools, such as big data analytics and artificial intelligence, will expedite research progress in this domain, foster the development of more globally competitive TCM products, and ultimately augment the role of traditional Chinese medicine in addressing obesity on a worldwide scale.

## Glossary

WHO: World Health Organization
TCM: Traditional Chinese Medicine
BMI: body mass index
WC: waist circumference
WHO: World Health Organization
NAFLD: nonalcoholic fatty liver disease
WHR: waist-hip ratio
CUN-BAE: Clinica Universidad de Navarra-Body Adiposity Estimator
CT: computed tomography
MRI: magnetic resonance imaging
IGFBP-1: insulin-like growth factor binding protein-1
GRS: genetic risk score
TNF-α: tumor necrosis factor-α
IL-6: interleukin-6
EDCs: endocrine disrupting chemicals
NF-κB: nuclear transcription factor-κB
25 (OH) D: 25-hydroxyvitamin D
FDA: Food and Drug Administration
GLP-1: glucagon-like peptide-1
LSG: laparoscopic sleeve gastrectomy
LRYGB: laparoscopic Roux-en-Y gastric bypass
RCTs: randomized controlled trials
TJ: Taeeumjowuitang
HOMA-IR: Homeostasis Model Assessment-Insulin Resistance Index
TC: total cholesterol
TG: triglyceride
FPG: fasting plasma glucose
FINS: fasting insulin
PPARγ: peroxisome proliferator-activated receptor-γ
NPY: neuropeptide Y
ADP: adiponectin
HDL-C: high-density lipoprotein cholesterol
